# scPCOR-seq enables co-profiling of chromatin occupancy and RNAs in single cells

**DOI:** 10.1038/s42003-022-03584-6

**Published:** 2022-07-08

**Authors:** Lixia Pan, Wai Lim Ku, Qingsong Tang, Yaqiang Cao, Keji Zhao

**Affiliations:** Laboratory of Epigenome Biology, Systems Biology Center, Division of Intramural Research, National Heart, Lung and Blood Institute, National Institutes of Health, Bethesda, MD USA

**Keywords:** Sequencing, Biotechnology

## Abstract

Cell-to-cell variation in gene expression is a widespread phenomenon, which may play important roles in cellular differentiation, function, and disease development^1–9^. Chromatin is implicated in contributing to the cellular heterogeneity in gene expression^10–16^. Fully understanding the mechanisms of cellular heterogeneity requires simultaneous measurement of RNA and occupancy of histone modifications and transcription factors on chromatin due to their critical roles in transcriptional regulation^17,18^. We generally term the occupancy of histone modifications and transcription factors as Chromatin occupancy. Here, we report a technique, termed scPCOR-seq (single-cell Profiling of Chromatin Occupancy and RNAs Sequencing), for simultaneously profiling genome-wide chromatin protein binding or histone modification marks and RNA expression in the same cell. We demonstrated that scPCOR-seq can profile either H3K4me3 or RNAPII and RNAs in a mixture of human H1, GM12878 and 293 T cells at a single-cell resolution and either H3K4me3, RNAPII, or RNA profile can correctly separate the cells. Application of scPCOR-seq to the in vitro differentiation of the erythrocyte precursor CD36 cells from human CD34 stem or progenitor cells revealed that H3K4me3 and RNA exhibit distinct properties in clustering cells during differentiation. Overall, our work provides a promising approach to understand the relationships among different omics layers.

## Abstract (web summary)

scPCOR-seq is a single-cell sequencing technique that enables simultaneous profiling of genome-wide chromatin protein binding or histone modification marks and RNA expression in the same cell.

## Introduction

Gene expression exhibits remarkable cellular heterogeneity, which may be influenced by multiple factors including different aspects of chromatin modifications^19–27^. In the past few years, several assays measuring different aspects of chromatin states at a single-cell resolution have been developed. These include Droplet-based single-cell ChIP-seq^28^, Tn5-based chromatin accessibility assays (ATAC-seq)^15,29^, DNase I hypersensitivity assay(DNase-seq)^10^, MNase-based nucleosome position and chromatin accessibility assay (scMNase-seq)^11^, immunocleavage-based histone modification assays (Cut&Run, scChIC-seq)^30–32^, antibody-guided Tn5 chromatin tagging assays (ACT-seq, Cut&Tag, CoBATCH)^33–35^, and NOMe-seq assay^36^. These assays measure one or more aspects of chromatin states and provided data on cellular heterogeneity in chromatin but do not directly measure simultaneously both RNA and chromatin in the same single cell.

To directly investigate the mechanisms that may regulate the cellular heterogeneity in gene expression, several laboratories have reported the techniques for co-profiling of both RNA and chromatin accessibility by combining single-cell RNA-seq and single-cell ATAC-seq^22,37,38^, providing powerful tools to investigate how chromatin accessibility contributes to cellular heterogeneity in the same single cell. Since chromatin states are controlled by multiple mechanisms including various histone modifications and chromatin binding proteins, it will be important to examine the RNA expression and histone modifications or chromatin binding profiles in the same cell.

## Results and discussion

### Experimental procedure

**Fig. 1:**
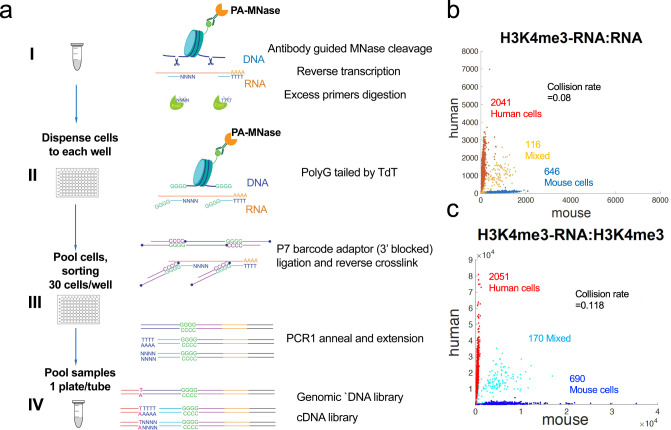
Description of the experimental steps of scPCOR-seq and its quality. **a** A schematic diagram showed the experimental steps of scPCOR-seq. **b** A scatter plot showing the number of reads that mapped to human and mouse genome for RNA reads. **c** A scatter plot showing the number of reads that mapped to human and mouse genome H3K4me3 reads.

We previously developed an indexing single-cell ChIC-seq protocol to profile histone modifications^39^ in which Terminal Transferase was used to mediate dG tailing on MNase digestion sites, meanwhile oligo-dC protruding barcode adaptors were ligated to these sites by T4 Ligase. Based on the above strategy, we developed scPCOR-seq (single-cell Profiling of Chromatin Occupancy and RNAs Sequencing), for simultaneously profiling genome-wide chromatin protein binding or histone modification marks and RNA expression in the same cell (Note that “chromatin occupancy” here refers to the bindings of histone modifications or DNA binding proteins). To capture both histone modification or DNA binding proteins on chromatin and RNA in the same cell, we devised a strategy to detect RNA profiles simultaneously (Fig. 1a). Briefly, Protein A-MNase (PA-MNase) was guided by specific antibodies to the targeted sites in formaldehyde-fixed cells. Following Ca^2+^-activated MNase digestion of chromatin, in-situ reverse transcription was performed by Maxima H Minus reverse transcriptase along with oligodT primer and a mixture of 749 not-so-random primers that do not recognize rRNAs (see “Methods” and Supplementary Data 1 for details). Then both the MNase-digested sites and cDNA were tailed simultaneously by Terminal Transferase and ligated with barcode adaptors in 96-well plate. The cells were pooled and sorted into a new 96-well plate with 30 cells per well by flow cytometry sorting, followed by two consecutive rounds of indexed PCR and final library sequencing. Single cells were resolved by identifying the unique combinations of barcodes and indexes as previously reported^15,29^.

### Profiling H3K4me3 and RNA in cell lines using scPCOR-seq

We first demonstrated the application of scPCOR-seq to profiling H3K4me3 and RNAs in human 293 T cells and mouse NIH 3T3 cells to estimate the detection of doublets. After identifying the barcodes that refer to cells in either RNA or H3K4me3 data, we observed a collision rate of 0.08 in the RNA data (Fig. 1b) and a collision rate of 0.118 in the H3K4me3 data (Fig. 1c). The different number of reads in RNA and H3K4me3 may have caused the discrepancy in the collision rate between H3K4me3 and RNA data. However, collision rates obtained in both data suggest that the doublets rate in scPCOR-seq is comparable to previous published single-cell assays^22,38,40^.

**Fig. 2:**
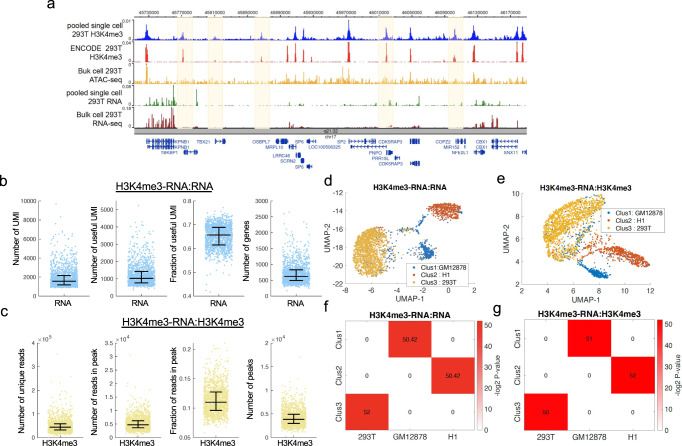
Co-Profiling H3K4me3 and RNA at single-cell level using H1, GM12878 and 293 T cells. **a** Co-Profiling H3K4me3 and RNA at single-cell level using 293 T cells. A genome browser snapshot showing five panels of data. From the top to the bottom, the first panel in blue shows the H3K4me3 profile of pooled (3717) single cells from the joint measurement of H3K4me3 and RNA using the scPCOR-seq assay. The second panel in red shows the bulk cell H3K4me3 profile of the bulk cell ChIP-seq data (SRR5627135) for 293 T cells. The third panel in yellow shows the bulk cell ATAC-seq profile (SRR5627157) for 293 T cells. The fourth panel in green shows the RNA profile of pooled (3713) single cells from the joint measurement of H3K4me3 and RNA using the scPCOR-seq assay. The fifth panel in red shows the bulk cell RNA-seq profile (SRR6504956) for 293 T cells. The highlighted regions show peaks detected by both H3K4me3 ChIP-seq and scPCOR-seq but not detected by ATAC-seq. **b** Dot plots showing measurement of four metrics for the RNA part of scPCOR-seq (*n* = 3713 single cells) The four metrics are Number of UMI, Number of useful UMI, Fraction of useful UMI, Number of genes detected. Middle line: mean; box limits, upper and lower quartiles. **c** Dot plots showing measurement of four metrics for the H3K4me3 part of scPCOR-seq (*n* = 3713 single cells). The four metrics are Number of unique reads, Number of reads in peaks, Fraction of reads in peaks, Number of peaks detected. Middle line: mean; box limits, upper and lower quartiles. **d** A UMAP plot showing the clusters of single cells using the RNA data from the H3K4me3-RNA scPCOR-seq assay. A multilayer Louvain clustering was applied to jointly cluster single cells from both RNA and ChIC parts. **e** UMAP plots showing the clusters of single cells using the H3K4me3 data from the H3K4me3-RNA scPCOR-seq assay. A multilayer Louvain clustering was applied to jointly cluster single cells from both RNA and ChIC parts. **f** A heatmap showing the overlap between the differential genes from different groups. Single cells were clustered into three groups. The differential expressed genes between cluster 1, cluster 2, and cluster 3 were denoted as “Clus 1”, “Clus 2” and “Clus 3” as shown in the labels on the *y* axis. The differential expressed genes between the RNA-seq of 293 T, GM12878 and H1 cells were denoted as “293 T”, “GM12878” and “H1” as shown in the labels on the *x* axis. The significance of overlap is determined by the hypergeometric test, which is shown by the color level (negative log of the *p* value). **g** A heatmap showing the overlap between the differential H3K4me3 peaks from different groups. Single cells were clustered into three groups. The differential H3K4me3 peaks between cluster 1, cluster 2, and cluster 3 were denoted as “Clus 1”, “Clus 2”, and “Clus 3” as shown in the labels on the *y* axis. The differential H3K4me3 peaks between the H3K4me3 ChIP-seq of 293 T, GM12878, and H1 cells were denoted as “293 T”, “GM12878”, and “H1” as shown in the labels on the *x* axis. The significance of overlap is determined by the hypergeometric test, which is shown by the color level (negative log of the *p* value).

Next, we first profiled H3K4me3 and RNAs by applying scPCOR-seq to a mixture of human H1 ESCs, 293 T cells, and GM12878 cells. After sequencing the libraries, the RNAs were distinguished from chromatin targets by a unique barcode embedded in the primers used for reverse transcription. In all, 3713 single cells were identified from the sequencing data (~2000 RNA UMI per cell and 45,000 H3K4me3 unique reads per cell, see Supplementary Data 2). Because measurement of the H3K4me3 signals on chromatin requires the cleavage of chromatin by pA-MNase recruited to the H3K4me3 target sites by the H3K4me3 antibodies, we first examined whether the detected H3K4me3 reads in the scPCOR-seq libraries are dependent on the specific antibody or simply reflect the chromatin accessibility measured by ATAC-seq. For this purpose, the H3K4me3 reads from the scPCOR-seq data from pooled 293 T single cells were compared to the public bulk 293 T cell H3K4me3 ChIP-seq data and ATAC-seq data. Overall, 10,868, 10,860, and 10,837 peaks were identified from the bulk cell H3K4me3 ChIP-seq data, ATAC-seq data and pooled single-cell H3K4me3 data from scPCOR-seq data, respectively. A comparison of these peaks revealed that while 46% of the H3K4me3 ChIP-seq peaks and 47% of the scPCOR-seq H3K4me3 peaks overlapped with the ATAC-seq peaks (Supplementary Fig. 1a, b), 73% of the scPCOR-seq H3K4me3 peaks overlapped with the bulk cell H3K4me3 ChIP-seq peaks^41^ (Supplementary Fig. 1c). The results indicate that the H3K4me3 data have a higher similarity to the H3K4me3 ChIP-seq data than the ATAC-seq data^41^ (Supplementary Fig. 1a–c). The H3K4me3 signals from the pooled 293 T single cells were also compared with the bulk cell H3K4me3 ChIP-seq data and bulk cell ATAC-seq data at a randomly selected genomic locus (Fig. 2a). As shown by the highlighted regions, the H3K4me3 signals are more like the H3K4me3 ChIP-seq signals than the ATAC-seq signals, consistent with the global analysis above. These results indicate that the H3K4me3 data from scPCOR-seq is specific and is not simply related to chromatin accessibility.

### Three cell types can be identified accurately based on scPCOR-seq data

The quality of the single-cell RNA-seq data was quantified by different metrics (Fig. 2b; Supplementary Data 3). A median of 1300 (0.65 in terms of fraction) useful UMI (i.e., UMI located within gene regions) were detected per single cell. A median of 700 genes was detected per cell. Similarly, four metrics were used to quantify the quality of H3K4me3 signals. A median of 5400 unique reads (0.12 in terms of fraction) per single-cell were detected within the peaks identified using ENCODE data. A median of 3000 peaks was detected per cell (Fig. 2c). These results indicate that scPCOR-seq can simultaneously detect histone modification and RNA levels simultaneously at a single-cell resolution. Next, to further validate the scPCOR-seq data, we tested whether the single-cell RNA data or the H3K4me3 data from the assays can separate cells to different clusters. First, the principal component analysis (PCA) was directly applied to the scPCOR-seq RNA and H3K4me3 data separately. UMAP was applied to the reduced dimensions for scRNA and scH3K4me3, separately. Finally, the software MolTi^42^ (multiplex-modularity with the adapted Louvain algorithm to cluster single cells using both RNA and H3K4me3 data. Single cells were separated into three clusters (Cluster 1 in blue, Cluster 2 in red, and Cluster 3 in orange) from each dataset (Fig. 2d, e). The clusters were annotated by comparing to the specifically expressed genes (Fig. 2f) or specific H3K4me3 peaks based on the ENCODE data (Fig. 2g). The data indicate that Cluster 1, Cluster 2, and Cluster 3 are H1, GM12878, and 293 T cells, respectively (Fig. 2f, g). Overall, both the RNA and H3K4me3 data from the scPCOR-seq assay can correctly separate different cell types from a mixture of cells.

### Profiling RNAPII and RNA in cell lines using scPCOR-seq

**Fig. 3:**
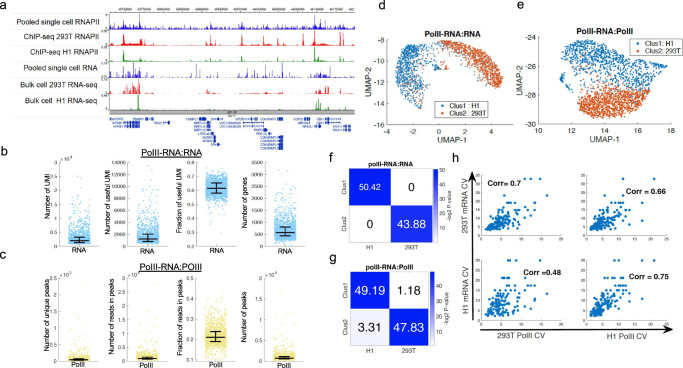
Co-Profiling PolII and RNA at single-cell level using H1 and 293 T cells. **a** A genome browser snapshot showing six panels of data. From the top to the bottom, the first panel in blue shows the RNAPII profile of pooled (2,347) single cells from the joint measurement of RNAPII and RNA using the scPCOR-seq assay. The second panel in red shows the bulk cell RNAPII profile of the bulk cell ChIP-seq data (SRR6927819) for 293 T cells. The third panel in green shows the bulk cell RNAPII profile of the bulk cell ChIP-seq data (SRR298998) for H1 cells. The fourth panel in blue shows the RNA profile of pooled (2347) single cells from the joint measurement of RNAPII and RNA using the scPCOR-seq assay. The fifth panel in red shows the bulk cell RNA-seq profile (SRR6504956) for 293 T cells. The sixth panel in green shows the ENCODE RNA-seq profile (ENCFF843FZA) for H1 ES cells. **b** Dot plots showing measurement of four metrics for the RNA part of scPCOR-seq (*n* = 2,347 single cells). The four metrics are Number of UMI, Number of useful UMI, Fraction of useful UMI, Number of genes detected. Middle line: mean; box limits, upper and lower quartiles. **c** Dot plots showing measurement of four metrics for the PolII part of scPCOR-seq seq (*n* = 2347 single cells). The four metrics are Number of unique reads, Number of reads in peaks, Fraction of reads in peaks, Number of peaks detected. Middle line: mean; box limits, upper and lower quartiles. **d** A UMAP plot showing the clusters of single cells using the RNA data from the PolII-RNA scPCOR-seq assay. A multilayer Louvain clustering was applied to jointly cluster single cells from both RNA and ChIC parts. **e** A UMAP plot showing the clusters of single cells using the PolII data from the PolII-RNA scPCOR-seq assay. A multilayer Louvain clustering was applied to jointly cluster single cells from both RNA and ChIC parts. **f** A heatmap showing the overlap between the differential genes from different groups. Single cells were clustered into two groups. The differential expressed genes between cluster 1, cluster 2 were denoted as “Clus 1” and “Clus 2 as shown in the labels on the *y* axis. The differential expressed genes between the RNA-seq of H1, and 293 T cells were denoted as “H1” and “293 T” as shown in the labels on the *x* axis. The significance of overlap is determined by the hypergeometric test, which is shown by the color level (negative log of the *p* value). **g** A heatmap showing the overlap between the differential PolII peaks from different groups. Single cells were clustered into two groups in Fig. 3d. The differential PolII peaks between cluster 1, cluster 2 were denoted as “Clus 1” and “Clus 2 as shown in the labels on the *y* axis. The differential PolII peaks between the PolII ChIP-seq of H1, and 293 T cells were denoted as “H1” and “293 T” as shown in the labels on the *x* axis. The significance of overlap is determined by the hypergeometric test, which is shown by the color level (negative log of the *p* value). **h** Four scatter plots between two variables (mRNA CV and PolII CV) at the cell type specific genes for 293 T and H1 cells.

To test whether scPCOR-seq can detect DNA binding proteins and RNAs in the same single-cell, we applied it to profiling both RNA Polymerase II (RNAPII) binding and RNAs in a mixture of H1 ESCs and 293 T cells. In addition, 2347 single cells were identified from the sequencing data (~3000 RNA UMI per cell and 7000 RNAPII unique reads per cell, Supplementary Data 3). The RNAPII binding and RNA signals from the pooled single cells were compared with ENCODE bulk cell RNAPII ChIP-seq data (Fig. 3a, top three tracks) and ENCODE RNA-seq data from H1 ESC and 293 T cells (Fig. 3a, bottom three panels), respectively. A median of 1900 (0.6 in terms of fraction) useful RNA UMI (i.e., UMI located within gene regions) were detected per single cell. A median of 700 genes were detected per cell (Fig. 3b, Supplementary Table 1). Also, four metrics were used to quantify the quality of RNAPII signals. A median of 1400 unique reads (0.2 in terms of fraction) were located within the peaks identified using ENCODE data. A median of 900 peaks were detected (Fig. 3c). These results indicate that scPCOR-seq can simultaneously detect faithfully RNAPII binding and RNA levels at a single-cell resolution. We used a similar strategy to cluster cells based on the RNA-RNAPII co-profiling data (Fig. 3d, e). Both the single-cell RNA and RNAPII occupancy data correctly clustered H1 and 293 T cells (Fig. 3f, g). Since RNAPII is directly responsible for producing RNAs and RNAPII binding from pooled single cells in H1 and 293 T cells indicates a positive correlation between RNAPII binding and RNA levels (Supplementary Fig. 1d, e), we next examined whether cell-to-cell variation in gene expression is correlated with that in RNAPII binding. The data indicate that cell-to-cell variation in gene expression is positively correlated with that in RNAPII binding in both H1 cells and 293 T cells (Fig. 3h). Importantly, this correlation is cell type specific meaning that the correlation is higher if both gene expression and RNAPII data are from the same cell type.

### Profiling H3K4me3 and RNA in human CD36 cells using scPCOR-seq

**Fig. 4:**
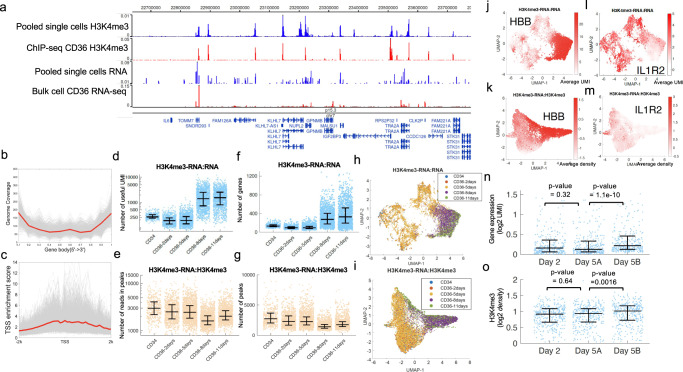
Co-Profiling H3K4me3 and RNA at single-cell level using CD34 and CD36 cells. **a** A genome browser snapshot showing four panels of data. From the top to the bottom, the first panel in blue shows the H3K4me3 profile of pooled single cells from the joint measurement of H3K4me3 and RNA using the scPCOR-seq assay. The second panel in red shows the bulk cell H3K4me3 profile of ChIP-seq data for CD36 cells. The third panel in blue shows the RNA profile of pooled single cells from the joint measurement of H3K4me3 and RNA using the scPCOR-seq assay. The fourth panel in red shows the bulk cell RNA-seq profile for CD36 cells. **b** A plot of Gene body coverage using the RNA data from scPCOR-seq data. The coverage of each single-cell is plotted in gray. The average coverage of single cells is plotted in red. **c** A plot of TSS enrichment profile for H3K4me3 data from scPCOR-seq data. The profile of each single-cell is plotted in gray. The average profile of single cells is plotted in red. **d** Dot plots showing the number of useful UMI of the RNA from scPCOR-seq. Middle line: mean; box limits, upper and lower quartiles. (*n* = 409 CD34 single cells, 871 CD36 2 days single cells, 1500 CD36 5 days single cells, 1500 CD36 8days single cells, 1500 CD36 11 days single cells). **e** Dot plots showing the number of genes recovered of the RNA from scPCOR-seq. Middle line: mean; box limits, upper and lower quartiles. **f** Dot plots showing the number of unique reads in peaks of the H3K4me3 from scPCOR-seq. Middle line: mean; box limits, upper and lower quartiles. **g** Dot plots showing the number of peaks of the H3K4me3 from scPCOR-seq. Middle line: mean; box limits, upper and lower quartiles. **h** A UMAP plot for scPCOR-seq that applied the RNA data in CD34 and CD36 cells. **i** A UMAP plot for scPCOR-seq that applied to the H3K4me3 data in CD34 and CD36 cells. **j** The gene expression level of HBB is shown in the UMAP plots from mRNA data. **k** The gene expression level IL1R2 is shown in the UMAP plots from mRNA data. **l** The H3K4me3 density of HBB is shown in the UMAP plots from H3K4me3 data. **m** The H3K4me3 density of IL1R2 is shown in the UMAP plots from H3K4me3 data. **n** Dot plots showing the expression of the genes, which are different between the Day 5 A group and Day 5B group cells (725 genes), in CD36 Day-2 cells, CD36 Day-5A cells, and CD36 Day-5B cells. Middle line: mean; box limits, upper and lower quartiles. (*n* = 866 CD36 Day-2 cells, 4202 CD36 Day-5A cells, 3272 CD36 Day-5B cells). *P* value is computed using the Wilcoxon rank sum test. **o** Dot plots showing the H3K4me3 density for genes in the Fig. 4n in CD36 Day-2 cells, CD36 Day-5A cells, and CD36 Day-5B cells. Middle line: mean; box limits, upper and lower quartiles. (*n* = 866 CD36 Day-2 cells, 4202 CD36 Day-5A cells, 3,272 CD36 Day-5B cells). *P* value is computed using the Wilcoxon rank sum test.

To test whether scPCOR-seq can be used to analyze more complex systems, we applied it to examining the in vitro differentiation of CD36+ erythrocyte precursor cells from human CD34+ hematopoietic stem/progenitor cells^43^. During the differentiation, the cell surface marker CD36 is significantly upregulated from day 5 and reaches peak expression by day 11, which is accompanied by decreased expression of CD34. We constructed libraries for both H3K4me3 and RNA for CD34+ cells and the cells differentiated for 2, 5, 8, and 11 days. Totally, 13,406 out of 14,167 single cells passed the QC (409 CD34 cells, 871 CD36 Day-2 cells, 7589 Day-5 cells, 3304 Day 8 cells, 1193 Day-11 cells). The H3K4me3 and RNA signals from the pooled single cells (CD36+ 11 days differentiation) were compared with the published bulk cell H3K4me3 ChIP-seq data (Fig. 4a, the second tracks counted from the top) and with the published bulk cell RNA-seq data from CD36+ cells (Fig. 4a, bottom track). From the genome coverage profile of the RNA-seq data, the reads are more likely to be located in the TSS and TES regions (Fig. 4b). The enrichment plot of H3K4me3 data (Fig. 4c) around TSS showed the average fold-enrichment of 2.5. The RNA part of scPCOR-seq showed lower complexity than the published^44^ bulk cell RNA-seq data using preseq^45^ (Supplementary Fig. 2a, b). To test whether the H3K4me3 signals are dependent on the H3K4me3 antibody, we compared the H3K4me3 signals from scPCOR-seq in CD36+ cells with the H3K4me3 ChIP-seq data and ATAC-seq data in CD36+ cells. As shown by the Genome Browser tracks at randomly selected genomic regions, the H3K4me3 data from scPCOR-seq data are more consistent with the H3K4me3 ChIP-seq data than the ATAC-seq data (Supplementary Fig. 2c). For the H3K4me3 data from scPCOR-seq, the average fraction of reads in peaks is ~0.4 while the average fraction of reads in peaks for H3K4me3 ChIP-seq data is ~0.8. For a global comparison between these different datasets, 21,290, 21,311, and 21229 peaks were identified from the bulk cell H3K4me3 ChIP-seq data, ATAC-seq data, and pooled single-cell H3K4me3 data from scPCOR-seq data, respectively. A comparison of these peaks revealed that while 72% of the H3K4me3 ChIP-seq peaks and 70% of the scPCOR-seq H3K4me3 peaks overlapped with the ATAC-seq peaks (Supplementary Fig. 2d, e), 86% of the scPCOR-seq H3K4me3 peaks overlapped with the bulk cell H3K4me3 ChIP-seq peaks (Supplementary Fig. 2f). The higher overlap between the H3K4me3 scPCOR-seq peaks with the H3K4me3 ChIP-seq peaks than that between the H3K4me3 scPCOR-seq peaks with the ATAC-seq peaks from both CD36+ and 293T cells (Supplementary Figs. 1a–c and 2d–f) suggests that the signal of scPCOR-seq is specific to the antibody.

For the RNA-seq data, the median of the useful UMI increased from CD34+ cells (~300 UMI) to CD36 cells at 11 days (~3000 UMI) (Fig. 4d). The number of detected genes also increased from CD34+ cells (~200 genes) to CD36+ cells at 11 days (~500 genes) (Fig. 4e). For the H3K4me3 data, the median of unique reads in peaks decreased from CD34+ cells (~12,000 unique reads) to CD36+ cells at 11 days (~7000 unique reads) (Fig. 4f). The number of detected peaks also decreased from CD34+ cells (~3000 peaks) to CD36+ cells at 11 days (~1200 peaks) (Fig. 4g). The different numbers in the metrics among the cells at different differentiation stages are possibly due to the differences in cellular environments (Supplementary Table 1). Next, the batch effects in the single cells were removed by FastMNN^46^ and projected into the reduced space from UMAP (Fig. 4h, i). We observed that the CD34+ cells and day 11 CD36+ cells were localized to two clusters that are most distant from each other in the plot with ether RNA or H3K4me3 data, which is consistent with the process of cell differentiation. The clusters of day 8 and day 11 CD36+ cells based on either RNA or H3K4me3 were very close to each other in the plot, indicating a high similarity between them. The day 2 CD36 cells exhibited high levels of heterogeneity in both the RNA and H3K4me3 plots, suggesting that the cells display heterogeneous levels of response to differentiation signals at the early stages of differentiation. Interestingly, the H3K4me3 data of day 5 CD36 cells displayed different patterns of clustering properties as compared to the RNA data. It was apparent that the day 5 CD36 cells based on the H3K4me3 data already exhibited a unique cluster that was localized between the clusters of CD34/CD36 (day 2) and CD36 (day 8 and 11) cells (Fig. 4i). However, clustering of the day 5 CD36 cells based on the RNA data separated the cells into two distinct clusters: one was localized between the clusters of CD34/CD36 (day 2) and CD36 (day 8 and 11) cells while the other was not separated from the CD34/CD36 (day 2) cells (Fig. 4h). These results potentially suggest that the changes in H3K4me3 may occur ahead of the changes in transcription during the differentiation process, implying that H3K4me3 plays a critical role in cell differentiation process which later controls the transcription landscape. Different cell type specific genes were selected (HBB is more specific in CD34 cells while IL1R2 is more specific in CD36 11 days). Their expression level and H3K4me3 density were shown in the UMAP spaces in which the change is also consistent to their cell-type specific roles (Fig. 4j–m).

As shown in Fig. 4h, i and Supplementary Fig. 2g, h, the cells at CD36 5 days were clustered into two groups using K-means method using the RNA data. The two clusters of cells were named as CD36 5 days-A and CD36 5 days-B (Supplementary Fig. 2g). The cells in CD36 5 days-A are more like CD34 cells and CD36 2 days cells (Supplementary Fig. 2g). Compared to Day 5 A cells, 341 genes have significant higher expression in Day 5B cells while no genes have lower expression in Day 5B cells (Fig. 4n). However, the H3K4me3 density at these genes do not show a significant increased H3K4me3 signals from Day 5A to Day 5B cells (Fig. 4o). This result may support that the changes in H3K4me3 occur ahead of the changes in transcription during the differentiation process.

## Conclusions

Elucidating cellular heterogeneity was shown to be important for understanding different biological processes, including cell differentiation and tumor progression etc. However, few studies addressed the question of origins and mechanisms of cellular heterogeneity in gene expression at a single-cell level. Several studies indicated variations in chromatin status may contribute to variations in gene expression, suggesting that both cis regulatory elements and trans acting chromatin binding factors play important roles in the cellular heterogeneity of gene expression. In this study, we developed scPCOR-seq, a method for simultaneously measuring RNA expression levels and chromatin occupancy of chromatin binding proteins or histone modifications in the same single-cell and demonstrated its application to human H1 ESCs, GM12878, and 293T cells. Profiling H3K4me3 and RNA by applying scPCOR-seq to human CD34+ and CD36+ cells revealed that both histone and RNA signals are cell-type specific, and H3K4me3 showed a change prior to the change of RNA in cells at CD36+ 5 days.

One limitation of the current scPCOR-seq protocol is that the RNA reads have relatively lower complexity and low genome overage rate compared to the bulk cell measurements. Proper clusters could be identified using the RNA part of the human CD36 scPCOR-seq data, but future studies should be careful about the interpretation of the RNA part of scPCOR-seq data. Overall, we conclude that scPCOR-seq will serve as a powerful tool to study the joint profiling of RNA and histone modification marks

## Methods

### Reagents

Histone H3 trimethyl Lys4 antibody was purchased from Millipore (catalog no. 07–473), RNAPII antibody was purchased from Abcam (catalog no. ab817). Methanol-free formaldehyde solution was purchased from Thermo Fisher Scientific (catalog no. 28906). Terminal Transferase was purchased from New England BioLabs (catalog no. M0315L). The human embryonic stem cell line H1 (WA01- lot WB35186 p30) was provided by WiCell Research Institute. PA-MNase was purified after transformation of PET15b-PA-MNase plasmid (Addgene#124883) into BL21 Gold (DE3) following standard protocol.

### Cell culture and fixation

HEK293T cells and GM12878 were maintained in DMEM (Invitrogen, catalog no. 10566-016) supplemented with 10% FBS (Sigma-Aldrich, catalog no. F4135-500ML) following standard procedure. The H1 human embryonic stem cell line was maintained in feeder-free mTeSR^TM^1 medium (Stem Cell Technologies, catalog no.85850) and passaged with ReLeSR^TM^ (Stem Cell Technologies, catalog no.05872) following the manufacturer’s instruction. Cells were harvested, washed with 1× PBS twice, and resuspended in DMEM containing 10% FBS and 1% formaldehyde. After 5 min incubation in room temperature, the reaction was stopped by adding 1.25 M glycine, followed by two rounds of washes with PBS. The cells were aliquoted into 1 × 10^6^ cells per tube, frozen on dry ice, and stored at −80°C.

### Antibody-guided MNase digestion and end repair

The fixed cells were thawed on ice. To prepare PA-MNase and antibody complex, 1 μl antibody and 3 μl PA-MNase were pre-incubated on ice in 4 μl antibody binding buffer (10 mM Tris-Cl (pH 7.5), 1 mM EDTA, 150 mM sodium chloride, 0.1% Triton X-100) for 30 min. Meanwhile, H1 fixed cells (1 million) and HEK 293 T fixed cells (1 million) were resuspended in 100 μl antibody binding buffer. Then, cell suspension was added to the PA-MNase and antibody complex, incubated on ice for 1 h. Cells were washed three times with high salt buffer (10 mM Tris-Cl (pH 7.5), 1 mM EDTA, 400 mM sodium chloride and 1% (v/v) Triton X-100), followed by washing once with rinsing buffer (10 mM Tris pH 7.5, 10 mM sodium chloride and 0.1% (v/v) Triton X-100). Then the cells were resuspended in 40 μl reaction solution buffer (10 mM Tris-Cl (pH 7.4), 10 mM sodium chloride, 0.1% (v/v) Triton X-100, 2 mM CaCl_2_), incubated at 37 °C for 3 min in water bath. The reaction was stopped by adding 4.4 μl 100 mM EGTA. After washing twice with rinsing buffer, the cells were end-repaired by T4 Polynucleotide Kinase (PNK) in 150 μl reaction buffer (1 x PNK buffer, 1 mM ATP, 150 unites PNK) at 37 °C for 30 min, followed by washing twice with rinsing buffer to stop the reaction.

### In-situ reverse transcription

The cells were resuspended in 25 μl reverse transcription buffer (5 μl 10 × Maxima H Minus reverse transcription buffer, 1.25 μl 10% NP40, 16.75 μl H_2_O, 1 μl 100 μm not-so-random primers mixture (Supplementary Data 1)^47^, 1 μl 10 ng/μl Oligo dT22 primer (NNNNNNGAGCGTTTTTTTTTTTTTTTTTTTTTTVN)). After incubated at 65 °C for 1 min, the reaction was immediately put on ice, while the enzyme mix is prepared (8.75 μl H_2_O, 5 μl 10× Maxima H Minus reverse transcription buffer, 8 μl 10 mM dNTPs, 2 μl Maxima H Minus reverse transcriptase, 0.625 μl SUPERase• In™ RNase Inhibitor, 0.625 μl RNaseOUT™ Recombinant Ribonuclease Inhibitor) and added into the reaction. The reverse transcription is performed as described in^37^ (50 °C × 10 min; 3 cycles for the following: 8 °C × 12 s, 15 °C × 45 s, 20 °C × 45 s, 30 °C × 30 s, 42 °C × 2 min, 50 °C × 5 min; 50 °C × 10 min and hold at 4 °C).

### Exonuclease I (Exo I) digestion

The cells were washed twice with rinsing buffer, resuspended in 50 μl reaction buffer (5 μl 10 × Exo I buffer, 1 μl Exo I, 44 μl H_2_O) and incubated at 37 °C for 20 min. This is to remove the excess primers left after reverse transcription. After digestion, the cells were washed twice with rinsing buffer to stop the reaction.

### Library construction

96 barcode-P7 adaptors (10 μM) stored in a 96-well plate were thawed at 4 °C, then 1 μl of each was added to the corresponding well in a new 96-well plate with multichannel pipette. Downstream library construction was performed same as the indexing single-cell ChIC-seq protocol^39^. Briefly, the cells were suspended with nuclei suspension buffer and mixed with enzyme dilution buffer, followed by aliquoted into 10 μl in 96 wells, mixing with the added barcode-P7 adaptors. The plate was sealed completely and incubated at 37 °C for 60 min. After incubation, the cells were pooled together in a solution trough containing 500 μl stop buffer, resuspended with 800 μl 1× PBS and send to flow cytometry core. 30 cells were sorted in each well of a new 96-well plate which contain 13 μl buffer mixture per well (3 μl reverse-crosslink buffer, 10 μl PBS containing 0.1% NP40). The plate was sealed completely and incubated at 65 °C for 6 h and 80 °C for 10 min.

After reverse-crosslink, indexed PCR1 was performed by adding 13 μl 2× Phusion® High-Fidelity PCR Master Mix with HF Buffer (New England BioLabs) and 1 μl 2 μM index primer with the following condition: 98 °C 3 min, 12 cycles of 65 °C 30 s, 72 °C 30 s, followed by 72 °C 5 min. Then the libraries were pooled together, digested with Exo I and purified by MinElute® Reaction Cleanup Kit (Qiagen). Downstream A-tailing and P5 adaptor ligation were performed same as the indexing single-cell ChIC-seq protocol^39^. PCR2 amplification with i5 index primer and P7-c2 primer was set in the following condition: 98 °C 3 min, 57 °C 3 min, 72 °C 1 min, 7 cycles of 98 °C 10 s, 65 °C 15 s, 72 °C 30 s, followed by 72 °C 5 min. The PCR products were run on the 2% E-Gel® EX Agarose Gel (Invitrogen). The fragments between 250–600 base pair (bp) were isolated and purified by the MinElute Gel Extraction Kit (Qiagen). The concentration of the library was measured by Qubit dsDNA HS kit (Thermo Fisher Scientific). The paired-end sequencing was performed on Illumina Hiseq 2500 and Novaseq.

### Data from other studies

ENCFF001RDI is for GM12878 RNA-seq. ENCFF843FZA is for H1 cells RNA-seq. SRR6504956 is for 293 T cells RNA-seq. SRR5627135 is for 293 T H3K4me3 ChIP-seq. ENCFF375WTP is for GM12878 H3K4me3 ChIP-seq. ENCFF285ZJI is for H1 cell H3K4me3 ChIP-seq. SRR5627157 is for 293 T cell ATAC-seq. SRR6927819 is for 293 T cell PolII ChIP-seq. SRR298998 is for H1 cell PolII ChIP-seq. SRR8509522 is for CD36 10 days ATAC-seq. SRR8358369 is for CD36 10 days H3K4me3 ChIP-seq. SRR8358300 is for CD36 11 days RNA-seq.

### Pre-processing of scPCOR-seq and Reads mapping

Pairs of reads were valid if read2 contain the exact linker sequences “AGAACCATGTCGTCAGTGT”. The valid pairs of read will be further separated into either RNA part or DNA part. If the linker sequences “GAGCG” for not-so-random primers or the linker sequences “CCTGCAGG” for oligodT were found in the location within 7–11th and 7–14th base of read1, the pair of reads were belonged to RNA. The remaining valid pairs were belonged to DNA. Using the information of the cell barcodes located at 5’ of read2, both pairs of reads belonging to RNA and DNA were separated into 96 sets of FASTQ files, respectively. Reads were mapped to the human reference genome hg19 using Bowtie2 Duplicates using different trimming parameters. Finally, the mapping results were combined, and Duplicated reads were removed based on mapping position and UMI for the reads belonging to DNA.

### Comparison of peaks

For scRNA-scH3K4me3 measurements using cell lines, cells were clustered and identified as 293T, H1, and GM12878 cells. The pseudo-bulk H3K4me3 ChIC-seq data from scRNA-scH3K4me3 measurements were pooled from the 293T cells. Also, we downloaded the H3K4me3 ChIP-seq data^41^ (SRR5627135), and ATAC-seq data (SRR5627157) for 293T cells. We used the software SICER to identify the similar number of peaks for each dataset: We identified 10,868 H3K4me3 ChIP-seq, 10,837 scPCOR-seq H3K4me3 peaks and 10,860 ATAC-seq peaks. Peak overlap was computed using BEDTools (bedtools intersect)^48^.

The pseudo-bulk scPCOR-seq H3K4me3 CD36 11 days data from scRNA-scH3K4me3 measurements were pooled. Also, we downloaded the H3K4me3 ChIP-seq data (SRR8358376) for CD36 11 days, and ATAC-seq data (SRR8509522) for CD36 cells 10 days. We used the software SICER to identify the similar number of peaks for each dataset: We identified 21,290 H3K4me3 ChIP-seq, 21,229 scPCOR-seq H3K4me3 peaks from our scPCOR-seq and 21,311 ATAC-seq peaks. Peak overlap was computed using BEDTools (bedtools intersect)^48^.

### Filtering for single cells and genes

For both scRNA-scRNAPII and scRNA-scH3K4me3 measurements using cell lines, genes and peak regions were excluded if less than 30 have reads in these regions. Single cells that have both at least 1000 RNA reads, and 1000 DNA reads were first considered. For both scRNA-scRNAPII, if single cells have reads in less than 100 peak regions or 100 gene regions, they were excluded. For both scRNA-scH3K4me3, if single cells have reads in less than 450 peak regions or 450 gene regions, they were excluded.

For scRNA-scH3K4me3 measurements using CD34 and CD36 cells, genes and peak regions were excluded if less than 30 have reads in these regions. If single cells have reads in less than 50 peak regions or 50 gene regions, they were excluded. Totally, 13,406 out of 14,167 single cells passed the QC.

### Dimension reduction for cell lines

For all scPCOR-seq measurements for cell line, the UMI matrix for RNA and the read count matrix for DNA were computed. The columns of RNA UMI matrix correspond to cells and its rows correspond to the genes. Similarly, the columns of DNA read count matrix correspond to cells and its rows correspond to the peak regions. The UMI matrix was transformed by based two logarithm transformations. The read count matrices were normalized by the library sizes and were transformed by based two logarithm transformations. For both RNA and DNA parts, PCA was applied to the two matrices. UMAP was further applied on the obtained principal component matrix.

### Cell clustering

For both RNA and DNA parts, cells were clustered for the scPCOR-seq cell line data. First, two cell-to-cell correlation matrices corresponding to RNA and DNA parts were computed using the obtained principal components. The *z* score transformation was applied to these matrices^49^. The edges between two genes/regions with *z* score values smaller than 3.2 were filtered out, resulting in two networks for RNA and DNA. The multiplex network clustering method MolTi^42^ was applied to both RNA and DNA networks.

### Removal of batch effect

scPCOR-seq CD34 and CD36 data were from four batches. The UMI matrix for RNA and the read count matrix for DNA were computed. The columns of RNA UMI matrix correspond to cells and its rows correspond to the genes. Similarly, the columns of DNA read count matrix correspond to cells and its rows correspond to the peak regions. For both RNA and DNA parts, batch effects were removed for the scPCOR-seq CD34 and CD36 data using FastMNN^46^. UMAP was further applied on the reduced matrix outputted by FastMNN^46^.

### Statistics and reproducibility

Hypergeometric test was used to calculate the *p* value in Figs. 2f, g, 3f, g. Two-sided Wilcoxon rank sum test was used to calculate the *p* value in Fig. 4n, o and Supplementary Fig. 1d, e. We jointly profiled scRNA-scPolII using 2000 mixed H1 and 293 T cells and scRNA-scH3K4me3 using 4000 mixed H1 293 T cells, GM12878. We jointly profiled scRNA-scH3K4me3 using a total of 14,167 sorted CD34, CD36 2 days, CD36 5 day, CD36 8 day, and CD36 11day cells. There are no replicates involved in each experiment. However, the three independent scPCOR-seq experiments support the robustness of the method.

### Reporting summary

Further information on research design is available in the Nature Research Reporting Summary linked to this article.

## Supplementary information

Supplementary Information

Description of Additional Supplementary Files

Supplementary Data 1

Supplementary Data 2

Supplementary Data 3

Reporting summary

## Data Availability

The scPCOR-seq data are available from GSE152057. The processed data can be downloaded from 10.6084/m9.figshare.19636866.v1.

## Code availability

The code can be downloaded from https://github.com/wailimku/scPCOR-seq.git.
