# Supplementary material for: scPCOR-seq enables co-profiling of chromatin occupancy and RNAs in single cells
Source: Commun Biol. 2022 Jul 8;5:678. doi: 10.1038/s42003-022-03584-6 (PMC9270334; doi:10.1038/s42003-022-03584-6)
Supplement: Supplementary file 1 — Supplementary Information [file 42003_2022_3584_MOESM1_ESM.pdf]

Supplementary Table 1

		PolII-mRNA	H3K4me3-mRNA	H3K4me3-mRNA	H3K4me3-mRNA	H3K4me3-mRNA	H3K4me3-mRNA	H3K4me3-mRNA
		cellline	celline	CD34	CD36-2days	CD36-5days	CD36-8days	CD36-11days
ChIC	Number of unique reads (median)	4633	43430	18632	14341	12644	5616	5615
ChIC	Number of unique reads in peaks (median)	1004	4694	2979	2501	2471	1608	2036
ChIC	Fraction of unique reads in peaks (median)	0.21	0.11	0.16	0.18	0.2	0.29	0.37
ChIC	Number of peaks (median)	743	3835	2734	2303	2261	1403	1749
RNA	Number of UMI (median)	1977	1562	438	344	342	2978	RNA
RNA	Number of useful UMI (median)	1198	1023	264	187	189	1403	1551
RNA	Fraction of useful UMI (median)	0.62	0.65	0.6	0.55	0.55	0.47	0.44
RNA	Number of genes (median)	564	621	132	96	96	274	334

Supplementary Table 1. Metrics for quantifying the quality of scPCOR-seq data.

Supplementary Figure 1

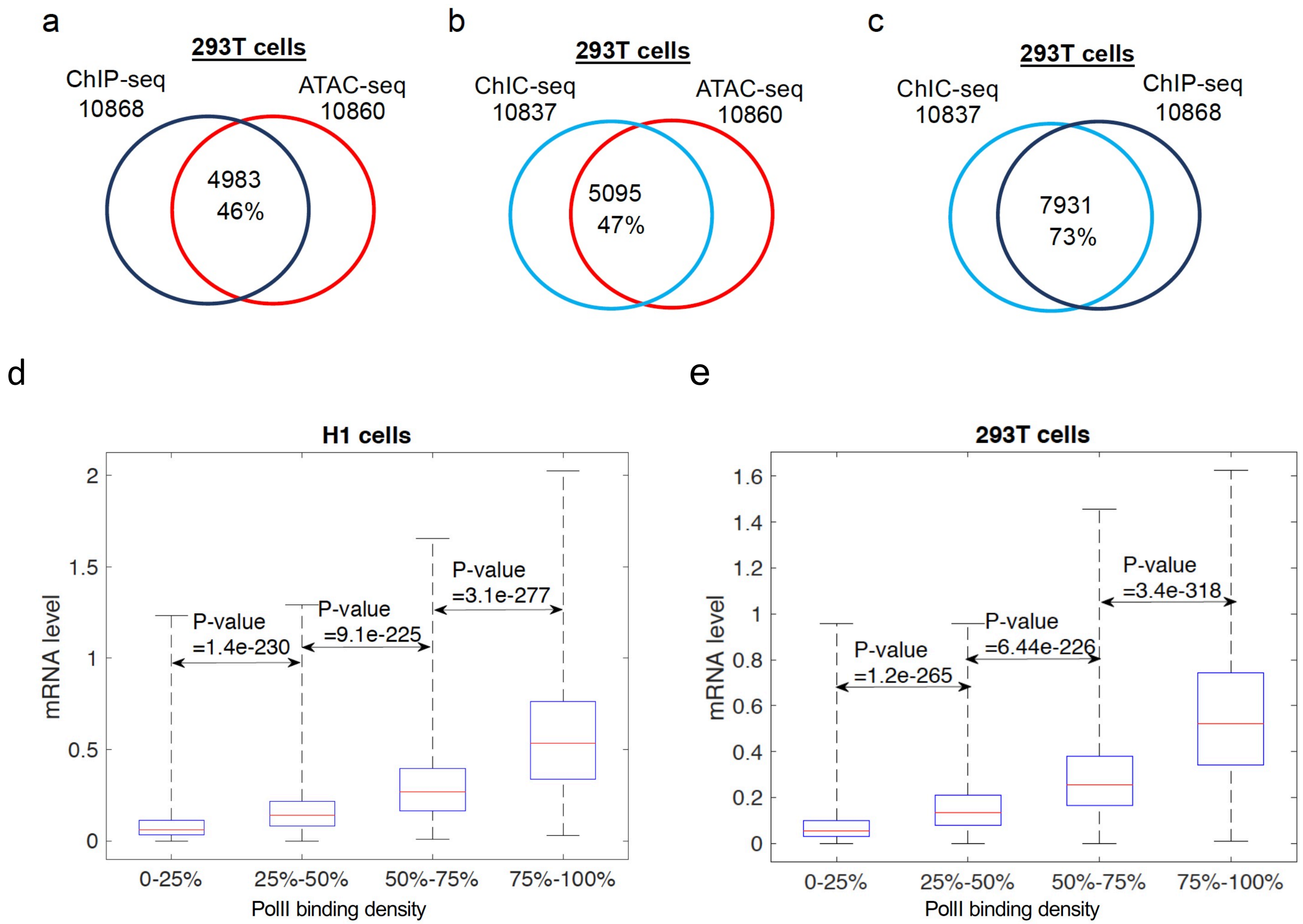

Co-Profiling H3K4me3/PolII and RNA at single cell level using cell line.

a. H3K4me3 ChIP-seq peaks is compared to ATAC-seq peaks for 293 T cells
b. scPCOR-seq H3K4me3 peaks is compared to ATAC-seq peaks for 293T cells.
c. H3K4me3 ChIP-seq is compared to scPCOR-seq H3K4me3 peaks for 293T cells.
d. For H1 cells, RNAPII binding is positively correlated with gene expression levels. Genes were separated into four groups based on the RNAPII binding levels in the pooled single cells (x-axis). The y-axis shows the RNA expression level of each group. Middle line: mean; box limits, upper and lower quartiles.
e. For 293T cells, RNAPII binding is positively correlated with gene expression levels. Genes were separated into four groups based on the RNAPII binding levels in the pooled single cells (x-axis). The y-axis shows the RNA expression level of each group.

Supplementary Figure 2

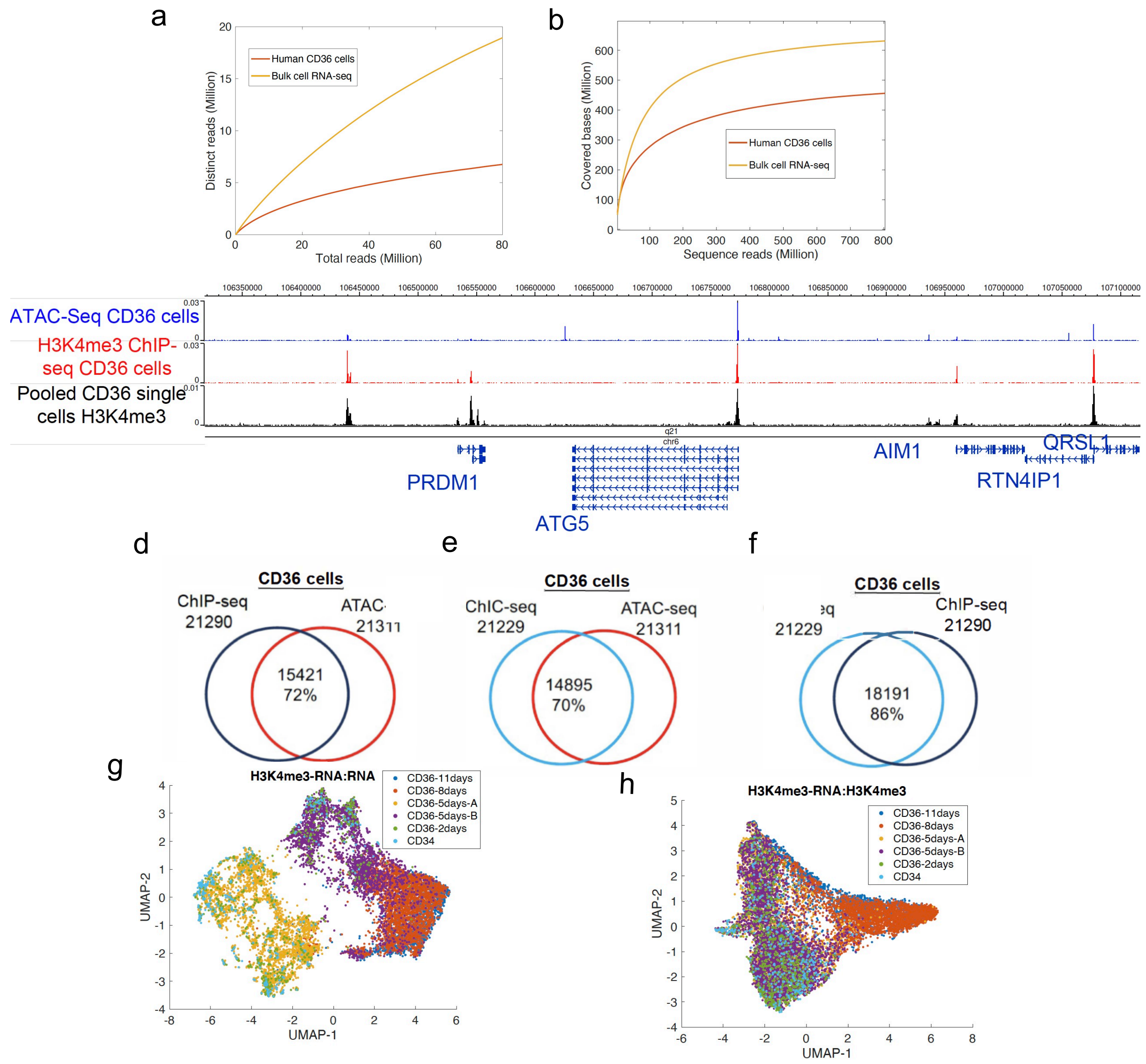

Co-Profiling H3K4me3 and RNA at single cell level using CD34 and CD36 cells.

a. A plot of distinct reads versus total reads generated for scPCOR-seq data of human CD36 cells (red), and Bulk cell RNA-seq data (yellow).
b. A plot of covered bases versus sequences reads generated for scPCOR-seq data of human CD36 cells (red), and Bulk cell RNA-seq data (yellow).
c. A genome browser snapshot showing three kinds of data in CD36 11 days cells. They are 1) ATAC-seq data, 2) H3K4me3 ChIP-seq data, and 3) H3K4me3 data from pooled single cell of scPCOR-seq data.
d. A Venn diagram for the comparison between H3K4me3 ChIP-seq peaks versus ATAC-seq peaks for CD36 11days.
e. A Venn diagram for the comparison between scPCOR-seq H3K4me3 peaks veersus ATAC-seq peaks for CD36 11days.
f. A Venn diagram for the comparison between H3K4me3 ChIP-seq versus scPCOR-seq H3K4me3 peaks for CD36 11days.
g. A UMAP plot showing the clusters of single cells using the RNA data from the PolII-RNA scPCOR-seq assay. K-means Method (k=2) was applied to the CD36 5days RNA data. CD36 5days cells were divided into two groups, CD36 5days-A and CD36 5days-B.
h. Similar to Supplementary Figure 2g, butplot showing the clusters of single cells using the PolII data from the PolII-RNA scPCOR-seq assay.
