# Supplementary material for: scPCOR-seq enables co-profiling of chromatin occupancy and RNAs in single cells
Source: Commun Biol. 2022 Jul 8;5:678. doi: 10.1038/s42003-022-03584-6 (PMC9270334; doi:10.1038/s42003-022-03584-6)
Supplement: Supplementary file 2 — Description of Additional Supplementary Files [file 42003_2022_3584_MOESM2_ESM.pdf]

Description of Additional Supplementary Files

File name: Supplementary Data 1
Description: The oligo sequences for 749 not-so-random primers.

File name: Supplementary Data 2
Description: Mapping Statistics for the scRNA-scH3K4me3 measurements.

File name: Supplementary Data 3
Description: Mapping Statistics for the scRNA-scRNAPII measurements.
