# Supplementary material for: scPCOR-seq enables co-profiling of chromatin occupancy and RNAs in single cells
Source: Commun Biol. 2022 Jul 8;5:678. doi: 10.1038/s42003-022-03584-6 (PMC9270334; doi:10.1038/s42003-022-03584-6)
Supplement: Supplementary file 6 — Reporting summary [file 42003_2022_3584_MOESM6_ESM.pdf]

nature research

Corresponding author(s): Keji Zhao

Reporting Summary

Nature Research wishes to improve the reproducibility of the work that we publish. This form provides structure for consistency and transparency in reporting. For further information on Nature Research policies, see Authors & Referees and the Editorial Policy Checklist.

Statistical parameters

When statistical analyses are reported, confirm that the following items are present in the relevant location (e.g. figure legend, table legend, main text, or Methods section).

n/a	Confirmed	
☐	☒	The exact sample size (n) for each experimental group/condition, given as a discrete number and unit of measurement
☐	☒	An indication of whether measurements were taken from distinct samples or whether the same sample was measured repeatedly
☐	☒	The statistical test(s) used AND whether they are one- or two-sided<br>*Only common tests should be described solely by name; describe more complex techniques in the Methods section.*
☒	☐	A description of all covariates tested
☐	☒	A description of any assumptions or corrections, such as tests of normality and adjustment for multiple comparisons
☐	☒	A full description of the statistics including central tendency (e.g. means) or other basic estimates (e.g. regression coefficient) AND variation (e.g. standard deviation) or associated estimates of uncertainty (e.g. confidence intervals)
☐	☒	For null hypothesis testing, the test statistic (e.g. F, t, r) with confidence intervals, effect sizes, degrees of freedom and P value noted<br>*Give P values as exact values whenever suitable.*
☒	☐	For Bayesian analysis, information on the choice of priors and Markov chain Monte Carlo settings
☒	☐	For hierarchical and complex designs, identification of the appropriate level for tests and full reporting of outcomes
☐	☒	Estimates of effect sizes (e.g. Cohen's d, Pearson's r), indicating how they were calculated
☒	☐	Clearly defined error bars<br>*State explicitly what error bars represent (e.g. SD, SE, CI)*

Our web collection on statistics for biologists may be useful.

Software and code

Policy information about availability of computer code

Data collection	No software was used for data collection.
Data analysis	Bowtie 2 (v2.2.5) was used for sequencing reads mapping. Ame was used to fine enriched motifs. Other data analysis was performed using custom codes and fully described in Methods. Custom codes for generating every steps of the analysis are available at https://github.com/wailimku/scPCOR-seq.git. The software Molti was used for cell clustering for cell line data. FastMNN was used for removing batch effects in CD34 and CD36 cells.

For manuscripts utilizing custom algorithms or software that are central to the research but not yet described in published literature, software must be made available to editors/reviewers upon request. We strongly encourage code deposition in a community repository (e.g. GitHub). See the Nature Research guidelines for submitting code & software for further information.

Data

Policy information about availability of data

All manuscripts must include a data availability statement. This statement should provide the following information, where applicable:

- Accession codes, unique identifiers, or web links for publicly available datasets
- A list of figures that have associated raw data
- A description of any restrictions on data availability

The scPCOR-seq data are available from GSE152057 , which can be accessed from the link below:
https://www.ncbi.nlm.nih.gov/geo/query/acc.cgi?acc=GSE152057

Field-specific reporting

Please select the best fit for your research. If you are not sure, read the appropriate sections before making your selection.

☒ Life sciences ☐ Behavioural & social sciences ☐ Ecological, evolutionary & environmental sciences

For a reference copy of the document with all sections, see nature.com/authors/policies/ReportingSummary-flat.pdf

Life sciences study design

All studies must disclose on these points even when the disclosure is negative.

Sample size	We jointly profiled scRNA-scPolII in the single cell level using scPCOR in 2,000 mixed H1 and 293T cells<br>We jointly profiled scRNA-scH3K4me3 in the single cell level using scPCOR in 4,000 mixed H1 293T cells, GM12878<br>The information of all libraries including libraries sizes, mapping statistics are included in Supplemental Data S2 and. Supplemental Data S3.<br>We jointly profiled scRNA-scH3K4me3 in the single cell level using scPCOR using 14,167 sorted CD34, CD36 2 days, CD36 5 day, CD36 8 day, and CD36 11 day cells
Data exclusions	For scRNA-scPolII and scRNA-scH3K4me3, we select the single cells that have reads more than 1000 RNA reads and 1000 DNA reads All information about data exclusion is included in the Supplemental Methods.
Replication	N/A
Randomization	For cell lines, single cells were randomly FACS sorted. For primary cells, we used sorted CD34, CD36 2 days, CD36 5 day, CD36 8 day, and CD36 11 day cells
Blinding	This study is in vitro experiment and no blinding is required. The investigators were not blinded during data collection and analysis.

Reporting for specific materials, systems and methods

Materials & experimental systems

n/a	Involved in the study	
☒	☐	Unique biological materials
☐	☒	Antibodies
☐	☒	Eukaryotic cell lines
☒	☐	Palaeontology
☒	☐	Animals and other organisms
☒	☐	Human research participants

Methods

n/a	Involved in the study	
☒	☐	ChIP-seq
☒	☐	Flow cytometry
☒	☐	MRI-based neuroimaging

Antibodies

Antibodies used	Histone H3 trimethyl Lys4 antibody were purchased from Millipore (catalog no. 07-473), Pol II antibody were purchased from

	Abcam (catalog no. ab817).
Validation	The qPCR is performed before library preparation for scPCOR-seq for both H3K4me3 Ab and polII Ab.

Eukaryotic cell lines

Policy information about cell lines

Cell line source(s)	HEK293T cells , H1 human embryonic stem cell line ,and GM12878 cells
Authentication	HEK293T cells and GM12878 cells were maintained in DMEM (Invitrogen, catalog no. 10566-016) supplemented with 10% FBS (Sigma-Aldrich, catalog no. F4135-500ML) following standard procedure. The H1 human embryonic stem cell line was maintained in feeder-free mTeSRTM1 medium (Stem Cell Technologies, catalog no.85850) and passaged with ReLeSRTM (Stem Cell Technologies, catalog no.05872) following the manufacturer's instruction.
Mycoplasma contamination	The cell lines were not tested for mycoplasma contamination.
Commonly misidentified lines (See ICLAC register)	No commonly misidentified cell lines were used.
